# Immune protection of three serine protease inhibitors vaccine in mice against *Rhipicephalus sanguineus*

**DOI:** 10.1038/s41598-024-58303-4

**Published:** 2024-04-02

**Authors:** Xiaoya Zhao, Jianguo Zhao, Jinhua Wang, Chenghong Liao, Qingfeng Guan, Qian Han

**Affiliations:** 1 Laboratory of Tropical Veterinary Medicine and Vector Biology, School of Life Sciences, Hainan University, Haikou, 570228 Hainan China; 2 One Health Institute, Hainan University, Haikou, 570228 Hainan China

**Keywords:** *R. sanguineus*, Serpins, Bioinformatics, Immune protection, Vaccine, Protein vaccines, Immunological techniques, Isolation, separation and purification, Molecular engineering

## Abstract

Bioactive molecules in tick saliva are considered to be key to successful feeding and further the transmission of tick-borne pathogens. Problems such as pathogen transmission and animal weight loss result in tick infestation can cause tremendous economic losses to the livestock industry. Therefore, the development of a universal tick vaccine is urgently needed. In this paper, three serine protease inhibitor (serpin) proteins RMS-3, L7LRK7 and L7LTU1 were analyzed with bioinformatics methods. Subsequently the proteins were expressed and purified, and inoculated into Kunming mice for immune protection analysis. The amino acid sequence similarities between RMS-3, L7LRK7 and L7LTU1 were up to 90% in *Rhipicephalus sanguineus*. The recombinant RMS-3 + L7LRK7 + L7LTU1 showed anticoagulant reaction function and could inhibit the activity of CD4^+^ lymphocytes, when inoculated into Kunming mice. Additionally, After the immunized mice were challenged with *Rhipicephalus sanguineus*, the percentage of larvae and nymphs that were fully engorged dropped to 40.87% (P < 0.05) and 87.68% (P > 0.05) in the RmS-3 + L7LRK7 immune group, 49.57% (P < 0.01) and 52.06% (P < 0.05) in the RmS-3 + L7LTU1 group, and 45.22% (P < 0.05) and 60.28% (P < 0.05) in the RmS-3 + L7LRK7 + L7LTU1 immune group, in comparison with the control group. These data indicate that RmS-3 + L7LRK7 + L7LTU1 has good immune protection and has the potential to be developed into a vaccine against the larvae and nymphs of *R. sanguineus*.

## Introduction

Ticks (Acari: Ixodida) belong to the class Arachnida^1^, and can be divided into three families: Argasidae (186 species), Ixodidae (692 species) and Nuttalliellidae (monotypic)^2^. So far, there are more than 900 known tick species in the world, of which the family Ixodidae account for more than 75% of tick species. Ticks are parasitic on the surface of vertebrates such as mammals for the most part, reptiles and birds, feeding on animal blood^3^, resulting in excessive blood loss, malnutrition, and even life-threatening of the animals. Ticks transmit a large number of pathogens, such as forest encephalitis virus, Crimean-Congo hemorrhagic fever virus, borrelia burgdorferi, etc., posing a great threat to the health of humans and animals^4–7^. Therefore, efficient prevention and control of ticks is overwhelmingly important to reduce the damage and losses they contribute to.

For a long time, ticks are mainly controlled by chemical acaricides, including pyrethroids, carbamates, organophosphates, organochlorines, formamidines, neonicotinoids, phenylpyrazoles, macrolides and benzoylphenylureas^8–11^. Nevertheless, excessive use of chemicals can give rise to environmental contamination and easily lead to tick resistance^12–14^. As an immunologic control approach, vaccines can be used as an important means of tick control due to their high specificity and innoxious to the environment^15–17^. In the early 1990s, a vaccine based on recombinant *Rhipicephalus microplus* intestinal antigen Bm86 was registered and marketed, and effectively reduced the number, weight and reproductive capacity of female ticks when applied to bovine hosts^18^. When feeding larval ticks a combination of anti-rBm86 and anti-RSUB (cytoplasmic Subolesin Antigen) antiserums, as opposed to anti-rBm86 antiserum alone, feeding of the ticks was greatly suppressed in a recent research on the *R. microplus* vaccine^19^. Moreover, many studies have shown that multi-antigen vaccination has a good effect on reducing tick infestation^20–22^. A cocktail vaccination that contains two or more antigens is therefore thought to act better against ticks.

With more than 249 proteins reviewed in uniprot database (https://www.uniprot.org/), serpins are one of the most well studied protease inhibitor families and are found in a wide range of protozoan, virus, mammals, and plant species^23^. Serpins have a role in the regulation of immunological responses, angiogenesis, clotting cascades, fibrinolysis, wound healing, and clotting in mammals^24^. Serpin sequences in arthropods, however, differ from those in mammals in a more significant way^25^. Additionally, research demonstrated that serpins served as immunosuppressants^26^, anti-complement proteins^27^, and anticoagulants^28^ in some insects. However, in blood-sucking arthropods such as ticks, serpins mainly function to prevent hemostasis and regulate immune response. It frequently exists in tick salivary glands, midgut, hemolymph and other tissues^29–31^, and was investigated as tick vaccines^32,33^. RMS-3 protein was a serpin first found in *R. microplus*^34^, regulating mast cells and showed an inhibitory effect on the metabolic activity of lymphocytes^35^ by inhibiting chymotrypsin and cathepsin G, as well as pancreatic elastase^36^, and was mainly expressed in the salivary glands of female adult ticks^37^. Nevertheless, further investigation on this protein’s immunoprotective properties is required.

In this study, bioinformatics was used to analyze the serpin RmS-3 and the putative tick salivary serpins L7LRK7 and L7LTU1 from *R. microplus*, which were predicted by sequencing^38^. The three proteins were then recombiantly expressed and purified, and immunoprotection of these proteins against *R. sanguineus* was analyzed, to confirm whether these proteins could be used as vaccine candidates for *R. sanguineus*.

## Methods

### Ticks and experimental animals

Kunming female mice, 6 weeks old, obtained from Hainan Pharmaceutical Research Institute. *R. sanguineus* used in the study were previously collected from the Stray Dog Center in Ding’an City (East longitude 110° 7ʹ–110° 14ʹ, north latitude 19° 33ʹ–19° 41ʹ), Hainan Province, China. They have since been reared in the laboratory in a climate controlled incubator at 27.5 °C, 89% relative humidity with 12 h light/dark cycles.

### In silico analysis

The amino acid sequences of RMS3 (GenBank number: AHC98654.1), L7LRK7 (GenBank number: JAA54167.1) and L7LTU1 (GenBank number: JAA54308.1) from *R. microplus* were blasted in Uniprot database (https://www.uniprot.org/), with the parametes of E-Threshold 10, and Auto-BLOSUM62 matrix, for the homology analysis of these proteins between different species.

The surface accessibility, flexibility and B cell epitope of RmS-3, L7LRK7 and L7LTU1 proteins was predicted and analyzed using DNAStar-protean software; T cell epitopes of RSM-3, L7LRK7 and L7LTU1 proteins were analyzed using AMPHI and Rothbard-Taylon algorithm in DNAstar-protean software.

### Expression and purification of recombinant proteins

The codons of RmS-3, L7LRK7, and L7LTU1 gene were optimized using the codon optimization tool of the online program ExpOptimizer (https://www.novopro.cn/tools/codon-optimization.html) in order to more effectively express the aforementioned proteins. The recombinant expression vector was created using the pTYB12 plasmid, and an *E. coli* BL21 (DE3) competent strain (Sango Biotech, China) was transformed with the recombinant plasmid.

Briefly, monoclonal strains were inoculated in Luria–Bertani (LB) broth at 37 °C on a platform shaker until an optical density at 600 nm (OD_600_) of 0.60 and subsequently induced for 24 h with 2 mM of Isopropyl b-d-1-thiogalactopyranoside (IPTG). The recombinant proteins were highly expressed and soluble. Bacteria pellets were resuspended in the lysis buffer (20 mM Tris–HCl (pH 8.5), 150 mM NaCl, 1 mM EDTA), ultrasonicated and centrifuged. The supernatant was purified with a Chitin (CBD-tag Affinity) Resin column (New England Biolabs, US). After purification, the fusion protein was cleaved at 16 °C or 23 °C for 24 h with a cleavage buffer (20 mM Tris–HCl (pH 8.5), 500 mM NaCl, 1 mM EDTA, 50 mM β-mercaptoethanol). Next, the target protein is eluted by the column buffer (20 mM Tris–HCl (pH 8.5), 500 mM NaCl, 1 mM EDTA). Finally, excess salt and β-mercaptoethanol are removed by desalting buffer. All proteins were determined by 12% sodium dodecyl sulfatepolyacrylamide gel electrophoresis (SDS–PAGE). The protein was then lyophilized with the ALPHA 1–2 LD plus freeze dryer (Christ, Germany) and preserved in − 20 °C.

### Vaccine formulation

Eight groups—RmS-3, L7LRK7, L7LTU1, RmS-3 + L7LRK7, RmS-3 + L7LTU1, L7LRK7 + L7LTU1, RmS-3 + L7LRK7 + L7LTU1 and control—were designed from 6-week-old female Kunming mice. Each group were vaccinated with corresponding antigens, while the control group were injected with PBS. Every group has 20 mice in it. The lyophilized protein was resuspended in sterile PBS to obtain a concentration of 1.0 mg/mL each. On days 0, 21 and 35, Kunming mice were subcutaneously injected, and the immunization dose of each mouse was 100 μL (25 μL antigen solution, 25 μL PBS solution and 50 μL adjuvant). 50 μL PBS solution and 50 μL adjuvant was used for injection to each animal in the control group. In order to achieve better immunization effect, complete Freund’s adjuvant (Sigma-Aldrich, Inc.) was used for the first immunization, and incomplete Freund’s adjuvant (Sigma-Aldrich, Inc.) was used for the remaining two immunizations. On the fourth day after each inoculation, serum was collected and stored at − 20 °C.

### Coagulation and routine analysis of blood

Orbital blood was collected from mice 7 days after the third immunization. The volume of blood collected from each mouse was 500 µL. Before detection, the blood was gently reversed and mixed for 10 times, and the blood routine was detected by automatic blood cell analyzer (BC-2800). The main detection indicators were as follows: white blood cell (WBC), lymphocyte (LYM), monocyte percentage (MON), neutrophil (NEU), red blood cell (RBC), hemoglobin (HGB) and platelet (PLT).

The clotting capability of the proteins RmS-3, L7LRK7, and L7LTU1 were measured by the following tests, prothrombin time (PT), activated partial thromboplastin time (aPTT), and thrombin time (TT). On the 4th day after each immunization, 3.2% sodium citrate anticoagulant was added to collect mouse blood. The blood samples were centrifuged at 3000 rpm for 10–15 min within two hours to obtain plasma, stored at − 20 °C, transported on dry ice, and sent to Servicebio company for testing.

### Antibody titer detection

A 96-well plate was coated with 0.1 μg/well recombinant protein in 100 μL carbonate/bicarbonate buffer (500 mM pH 9.6) and incubated overnight at 4 °C. The plate was washed three times (5 min each) with PBST (PBS containing 0.05% Tween-20) and then incubated with blocking solution (PBST containing 5% BSA) at 37 °C for 2 h. After washing with PBST three times, the plate was added with A series of 1:3 dilutions of mice serum and incubated for 1 h at 37 °C and washed as above. After that, 1 h incubation at 37 °C with 100 μL of HRP-conjugated goat anti-mouse IgG (diluted with 1:5000). The plate was washed again, and incubated with 100 μL of 3,3ʹ,5,5ʹ-tetramethylbenzidine (TMB) for 15 min at room temperature in the dark. 2 M H_2_SO_4_ (50 μL/well) was added for the end of the reaction, and the optical density (OD) of the product was measured at 490 nm by a microplate reader (Perlong Medical, Beijing). The OD450 nm (test group)/OD450 nm (negative control) ratio ≥ 2.1 was considered as a positive result.

### Identification of salivary proteins in *R. sanguineus*

Immunized mice serum was utilized for the detection of proteins RmS-3, L7LRK7 and L7LTU1 in *R. sanguineus* salivary glands. The salivary glands of 10 partially engorged female ticks were collected in a centrifuge tube containing 200 μL of carbonate/bicarbonate buffer (500 mM, pH 9.6), crushed and diluted to 20 mL as an antigen coating solution for iELISA detection. The operation refers to the description in “Antibody titer detection” section.

### Detection of CD4+, CD8+ T lymphocyte ratio

Two weeks after the last immunization, the mice spleen lymphocyte cells were harvested. In short, the mice were euthanized by cervical dislocation and soaked in 75% alcohol for 10 min for sterilization. Spleens were subsequently aseptically removed and a single cell suspension were prepared in PBS after lysis of red cells with adjustment of the cell concentration to 1 × 10^6^ cells/100 μL.

The lymphocytes isolated above were incubated with fluorescently labeled antibodies (FITC anti-mouse CD3ε, PE anti-mouse CD4, APC anti-mouse CD8a) at 4 °C for 50 min in the dark. One blank control tube (only cells, without any dye staining) and 3 single-dye adjustment compensation tubes (1 tube is only dyed with FITC dye, 1 tube is only dyed with PE dye, and the other tube is stained with APC dye only) were used as controls. Cells were washed twice with PBS and then resuspended in 200 μL of *PBS*. Flow cytometer (Beckman Coulter, USA) was used for detecting levels of T lymphocyte subgroups (CD4^+^ and CD8^+^).

### Challenge trials

On day 14 after the last immunization, tick challenge trial were performed on the immunized mice. The device for tick-infested mice was previously described^39^. For the tick challenge, there were 6 mice in each group, 3 mice were parasitized with 50 larval ticks per mouse; 3 mice were parasitized with 30 nymphs per mouse. The ticks were collected at 7 days after challenge, observe and count the changes in the tick weight, the number of engorged ticks and tick molting rate.

### Data analysis

All data were analyzed using GraphPad Prism 6.02 software. One-way ANOVA or two-way ANOVA with Dunnett’s multiple comparison test was performed for statistical analysis. The differences were considered statistically significant when *P* < 0.05.

### Approval for animal experiments

All animals used in the experiments were housed at the Vector Biology Laboratory, School of Life Sciences, Hainan University. The care and use of animals in this study were approved by the Hainan University Institutional Animal Use and Care Committee. The study is reported in accordance with ARRIVE guidelines. All the methods in this study were carried out in accordance with relevant guidelines and regulations.

## Result

### Sequence alignment and epitope analysis

**Figure 1:**
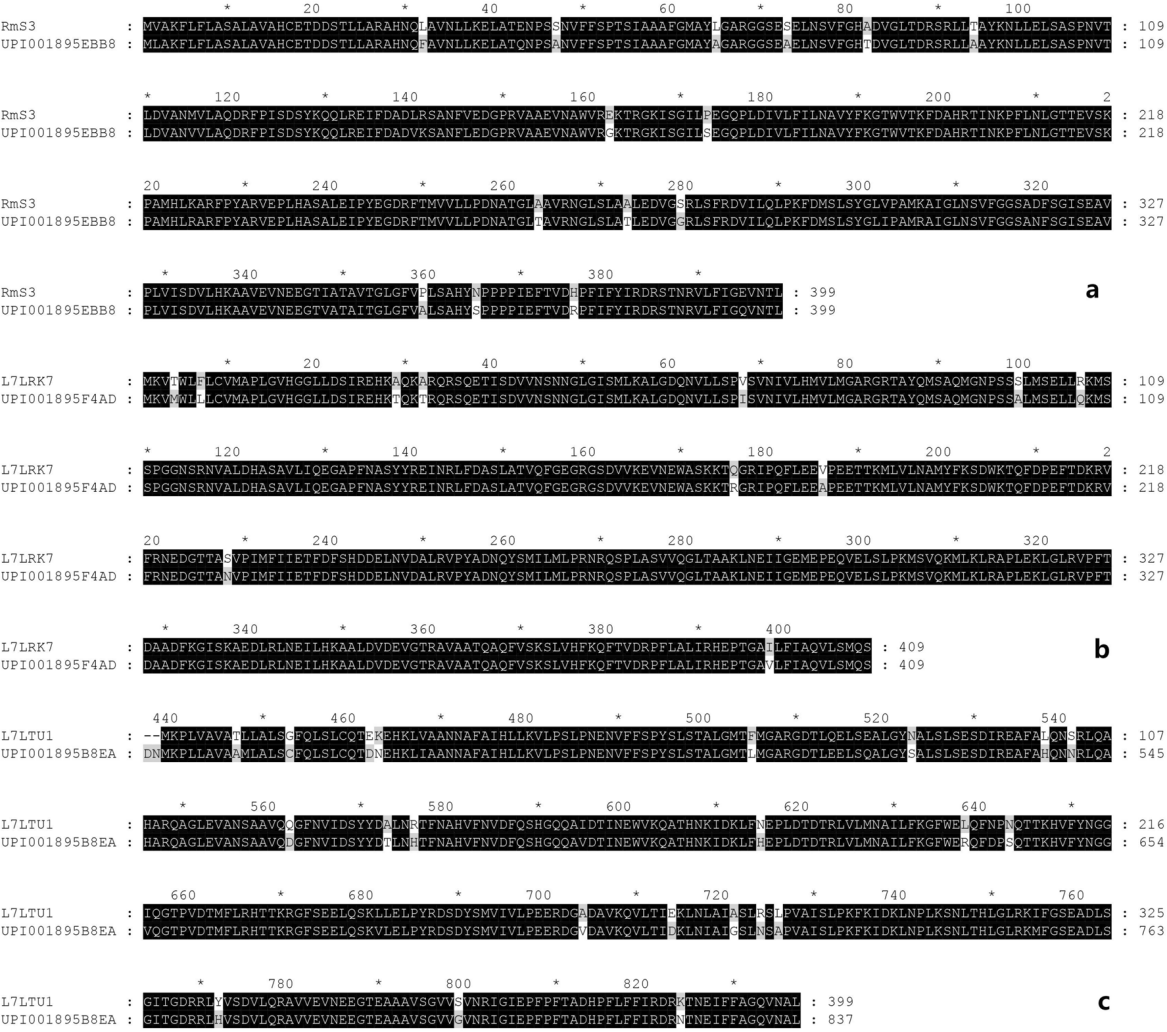
Alignment of the amino acid sequences RmS-3, L7LRK7 and L7LTU1 with the protein sequences RSS1 (Uniref entry UPI001895EBB8), RSS2 (Uniref entry UPI001895F4AD), and RSS3 (Uniref entry UPI001895B8EA) from *R. sanguineus,* respectively. Dark and grey indicates regions with identity of 100% and 80%, respectively. (**a**) RmS-3; (**b**) L7LRK7; (**c**) L7LTU1.

High similarity (identity > 90%) of serpins RmS-3, L7LRK7, and L7LTU1 was observed between *R. microplus* and *R. sanguineus* (UPI001895EBB8, UPI001895F4AD, and UPI001895B8EA in the Uniprot database; Fig. 1). Meanwhile, RmS-3, L7LRK7 and L7LTU1 share greater than 90% identity in *Rhipicephalus* spp., indicating that the three proteins were well conserved in *Rhipicephalus* spp.

The results of B cell epitope analysis were shown in Supplementary Fig. 1. There are 15 B cell antigen epitope fragments in RmS-3, 14 in L7LRK7, and 10 in L7LTU1. AMPHI algorithm and Rothbard-Taylor algorithm were used for predicting T-cell epitope of the three proteins. The comprehensive results of the two algorithms revealed that the T-cell epitope fragments were mainly residues 134–141, 158–165 in RmS-3, residues 38–45 in L7LRK7, and residues 134–141, 158–165 in L7LTU1. The presence of several epitopes suggested that these proteins might be immunogenic.

### Production of recombinant RmS-3, L7LRK7 and L7LTU1

**Figure 2:**
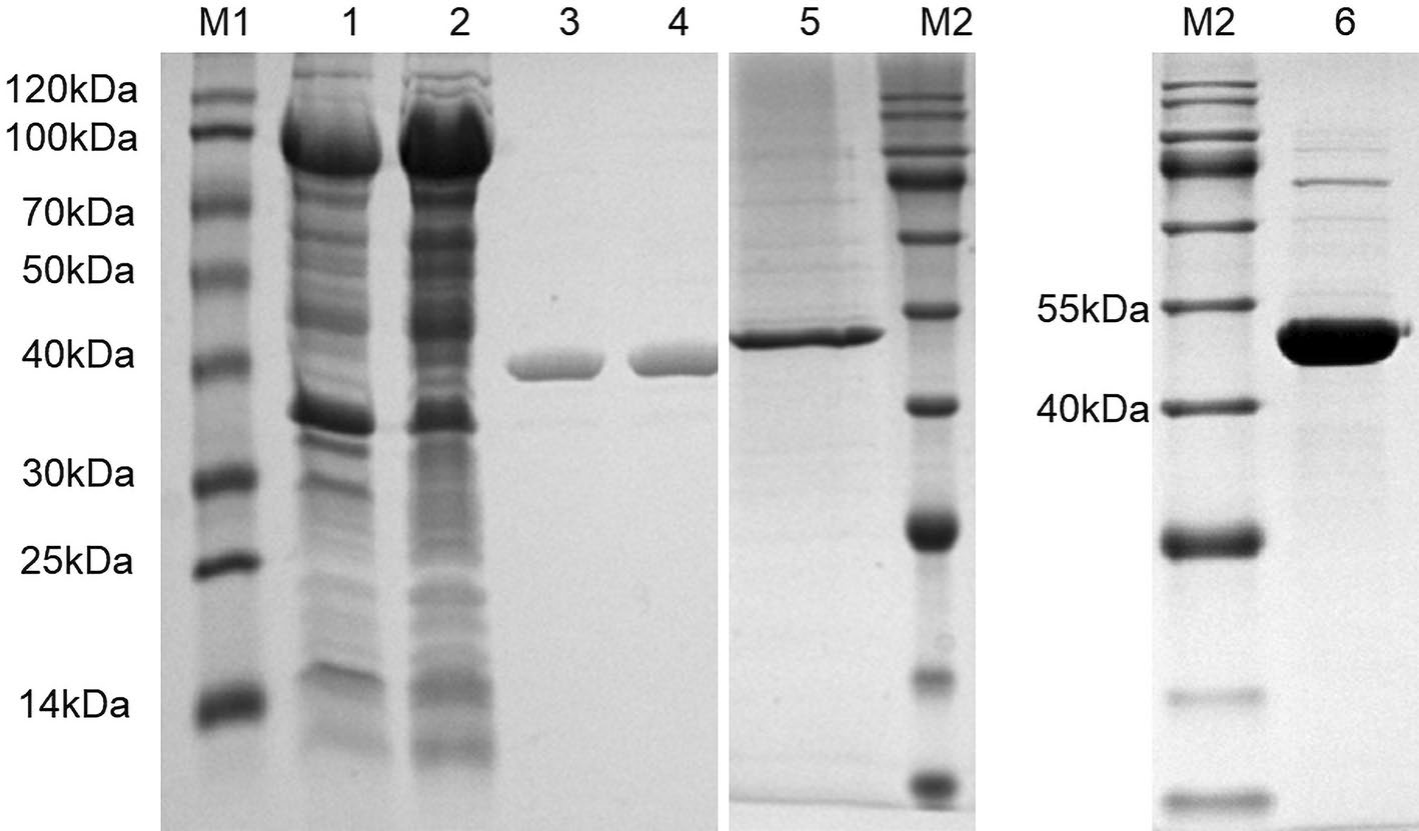
Expression and purification of rRmS-3, rL7LRK7 and rL7LTU1 analyzed by SDS-PAGE. Lane M1 and M2, protein marker. Lane 1 and 2, the crude extract from cell. RmS-3-CBD fusion proteins were expressed in *Escherichia coli*, isolated from cell lysates, and bound to a chitin-affinity column. Chitin binding domain-intein-RmS-3 fusion protein (~ 98 kDa); CBD, chitin binding domain (55 kDa). Lanes 3 and 4, the RmS-3 (~ 43 kDa). Lane 5, the L7LRK7 (~ 45 kDa). Lane 6, the L7LTU1 (~ 44 kDa).

The recombinant proteins RmS-3, L7LRK7 and L7LTU1 were expressed using the pTYB12 vector. The recombinant proteins of RmS-3, L7LRK7 and L7LTU1 with molecular weights of 43 kDa, 45 kDa and 44 kDa, respectively were obtained by Chitin Resin affinity chromatography (Fig. 2). The full length membranes of gel images were showed in Supplementary Figs. 2–4. Eventually, the purified proteins were dried in a vacuum freeze dryer at − 45 °C and stored at − 20 °C.

### Coagulation and immunity

Since serpins perform an important function in modifying the host immune response and coagulation cascade, they were validated by routine blood tests. In comparison with the control group, there was no significant difference in hematological indexes such as MON, NEU, RBC, HGB and PLT in the immune group. Nevertheless, except for the slight increase in RmS-3 immunized group, WBC and LYM in the other immunized groups showed a downward trend, and the WBC and LYM of L7LRK7 + L7LTU1 immunized group were significantly lower than those in the control group (Table 1).

**Table 1:** Blood test values after the third immunization of mice.

Blood test items	Control (n = 5)	RmS-3 (n = 5)	L7LRK7 (n = 5)	L7LTU1 (n = 5)	RmS-3/L7LRK7 (n = 5)	RmS-3/L7LTU1 (n = 5)	L7LRK7/L7LTU1 (n = 5)	RmS-3/L7LRK7/L7LTU1 (n = 5)	Reference value
WBC (10^9^/L)	6.24 ± 1.55	7.54 ± 0.91	4.81 ± 0.83	5.42 ± 2.26	4.95 ± 0.99	4.71 ± 1.27	3.99 ± 1.04*	4.43 ± 0.40	6.00–15.00
LYM (10^9^/L)	5.52 ± 1.35	6.58 ± 0.84	4.23 ± 0.90	4.36 ± 1.51	4.01 ± 0.84	3.92 ± 1.64	3.37 ± 0.86*	3.82 ± 0.28	3.40–7.44
MON (10^9^/L)	0.20 ± 0.05	0.25 ± 0.08	0.14 ± 0.08	0.27 ± 0.18	0.15 ± 0.08	0.17 ± 0.06	0.17 ± 0.08	0.17 ± 0.02	0.00–0.60
NEU (10^9^/L)	0.52 ± 0.23	0.70 ± 0.31	0.44 ± 0.15	0.79 ± 0.68	0.80 ± 0.20	0.62 ± 0.35	0.44 ± 0.15	0.44 ± 0.17	0.50–3.80
RBC (10^12^/L)	9.23 ± 1.07	9.07 ± 0.85	9.56 ± 0.38	9.85 ± 0.68	9.71 ± 0.37	10.00 ± 0.29	9.36 ± 0.49	9.05 ± 1.36	7.00–12.00
HGB (g/dL)	12.18 ± 1.43	11.74 ± 1.22	12.64 ± 0.50	12.38 ± 0.61	12.66 ± 0.48	12.82 ± 0.24	12.48 ± 0.54	11.88 ± 1.86	12.20–16.20
PLT (10^9^/L)	290.00 ± 36.91	303.80 ± 44.77	309.00 ± 11.38	327.20 ± 29.56	296.60 ± 29.60	306.60 ± 21.27	285.60 ± 46.20	329.20 ± 46.59	200.00–450.00

*WBC* white blood cell, *LYM* lymphocyte, *MON* monocyte percentage, *NEU* neutrophil, *RBC* red blood cell, *HGB* hemoglobin (), *PLT* platelet.

*(*P* < 0.05): significant difference between immune group and control group; *ns* no significant difference between immune group and control group, the same below.

**Figure 3:**
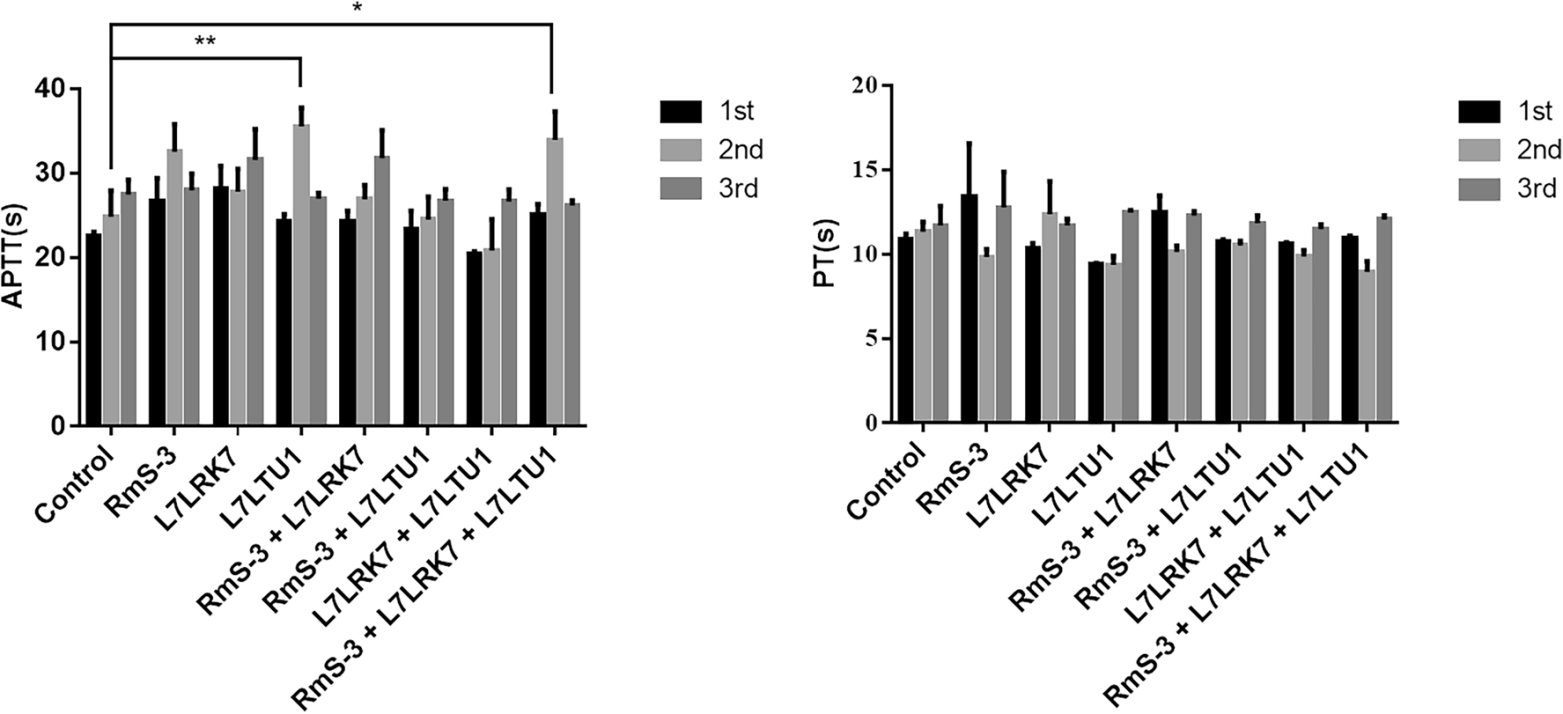
Coagulation reaction results. (**a**) Prothrombin time (PT) of blood in different groups. (**b**) Activated partial thromboplastin time (APTT) of blood in different groups; Abscissa: the immunization group that was the source of plasma.

Prothrombin time (PT) and activated partial thromboplastin time (APTT) of each group were measured to confirm the function of the serpins in the coagulation cascade. PT index data revealed no significant difference between the vaccinated and control groups. Furthermore, in compared to the control group, APTT of L7LTU1 and RmS-3 + L7LRK7 + L7LTU1 in the inoculated group was considerably extended after the second immunization (Fig. 3). These findings from the immunological groups L7LTU1 and RmS-3 + L7LRK7 + L7LTU1 imply that serpins have a role in blood coagulation inhibition.

**Figure 4:**
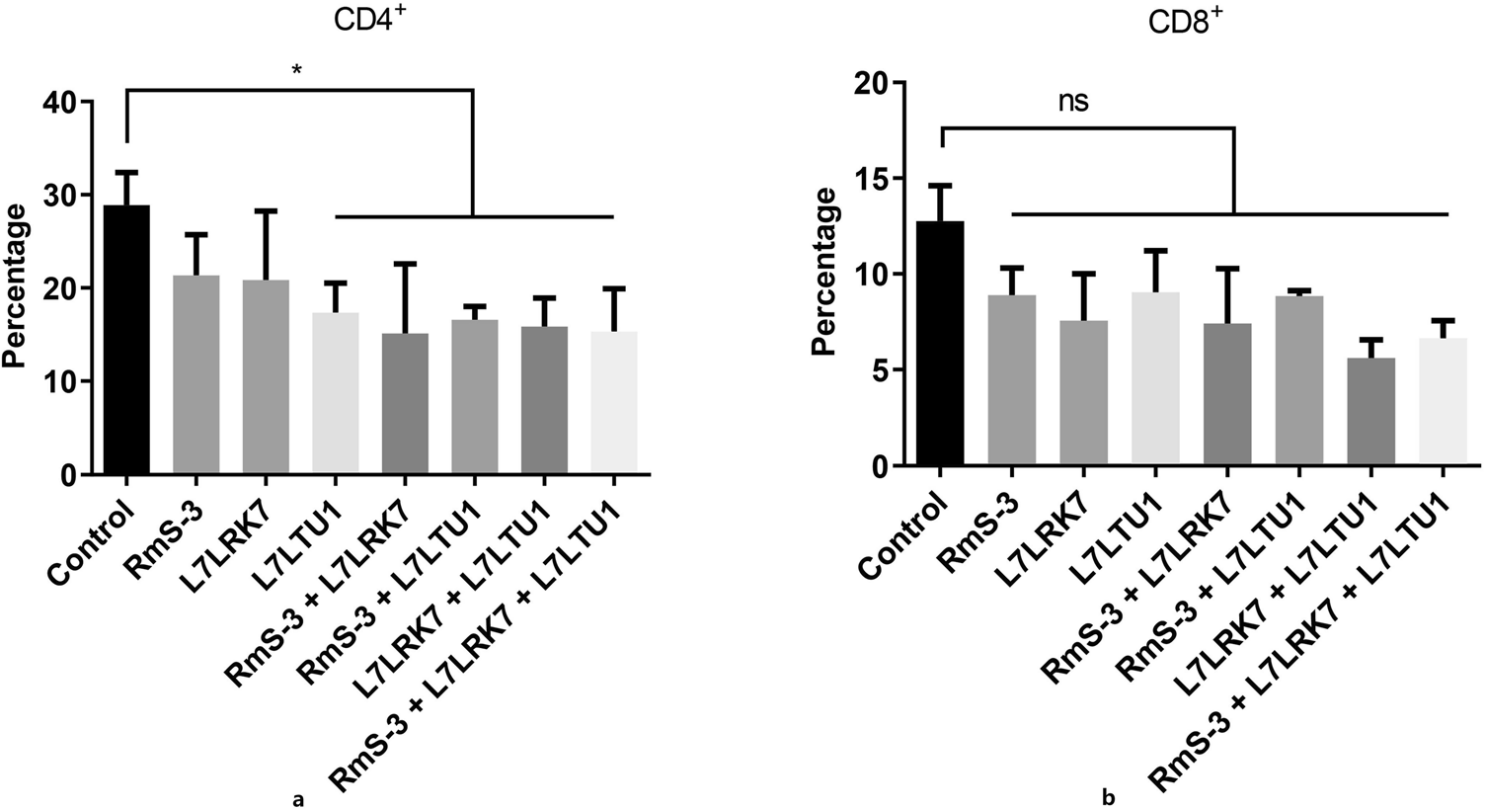
The levels of CD4^+^ and CD8^+^ T lymphocyte subsets in different groups after immunization of mice. (**a**) The levels of CD4^+^T lymphocyte subsets. (**b**) The levels of CD8^+^. Ordinata: percentage of CD4^+^ and CD8^+^ T lymphocytes in total T lymphocytes; Abscissa: spleen lymphocytes of mice in each group after immunization.

After the third vaccination of mice, the percentage of CD4+ and CD8+ spleen cells was determined by flow cytometry. Figure 4 illustrates how the proportion of CD4^+^ and CD8^+^ cells in the vaccinated group usually reduced when compared to the control group. The percentage of CD4^+^ cells in the vaccinated groups L7LTU1, RmS-3 + L7LRK7, RmS-3 + L7LTU1, L7LRK7 + L7LTU1, and RmS-3 + L7LRK7 + L7LTU1 decreased to 59.98%, 15.3%, 57.32%, 19.14%, and 10.50 (P < 0.05) compared to the control group. There was no discernible difference between the vaccinated and control groups (P > 0.05), despite the fact that the proportion of CD8+ cells in the immunized groups was lower than that in the control group. Following the third mouse vaccination, the results of a routine blood test revealed that the WBC and LYM levels in the L7LRK7 + L7LTU1-immunized group were considerably lower than those in the control group.

### Serum antibody titers

**Figure 5:**
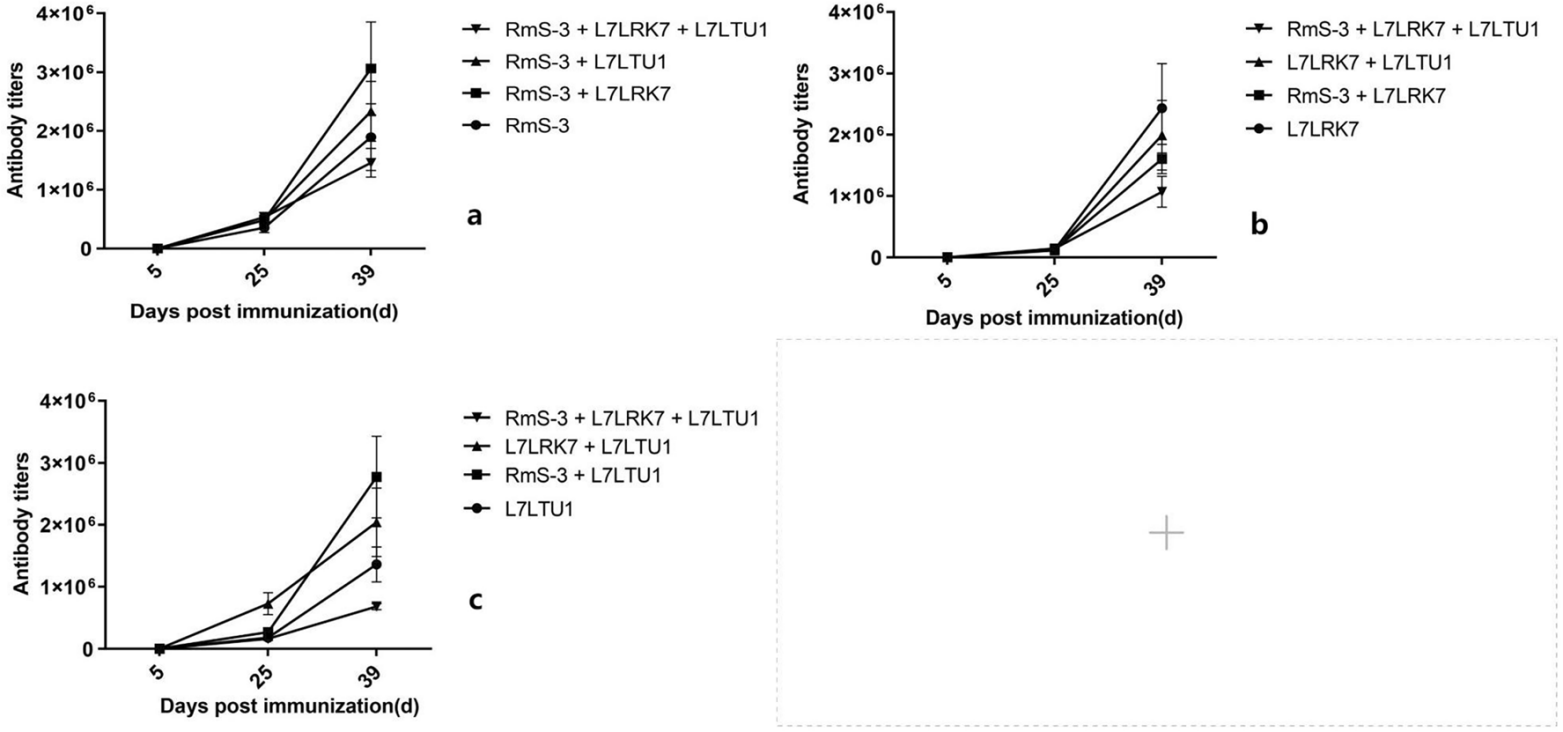
The antibody titers of mouse sera in different groups after immunization. (**a**) RmS-3 coated 96 well plates; (**b**) L7LRK7 coated 96 well plates; (**c**) L7LTU1 coated 96 well plates; Ordinata: antibody titer; Abscissa: blood collection time after immunization of mice.

The 96-well microtiter plate was coated with RmS-3, L7LRK7 or L7LTU1 antigens respectively, and the serum antibody titers of the immune groups were detected by iELISA. The effect of single-antigen vaccine and mixed-antigen vaccine was tested by detecting the antibody titers of anti-RmS-3 in the sera of RmS-3, RmS-3 + L7LRK7, RmS-3 + L7LRK7 + L7LTU1 immune groups. The results (Fig. 5) showed that the overall antibody titers were lower than 1000 after the first immunization, and the antibody titers gradually increased after the second and third immunizations. When the antigen was coated with RmS-3, the antibody titer was as follows: RmS-3 + L7LRK7 > RmS-3 + L7LTU1 > RmS-3 > RmS-3 + L7LRK7 + L7LTU1 in the immune groups; When antigen-coated with L7LRK7, the antibody titer results were L7LRK7 > L7LRK7 + L7LTU1 > RmS-3 + L7LRK7 > RmS-3 + L7LRK7 + L7LTU1 in the immune group; When the L7LTU1 protein was used for antigen coating, the antibody titer was as follows: RmS-3 + L7LTU1 > L7LRK7 + L7LTU1 > L7LTU1 > RmS-3 + L7LRK7 + L7LTU1 in immune groups.

### Identification of RmS-3, L7LRK7 and L7LTU1 proteins in *R. sanguineus*

**Figure 6:**
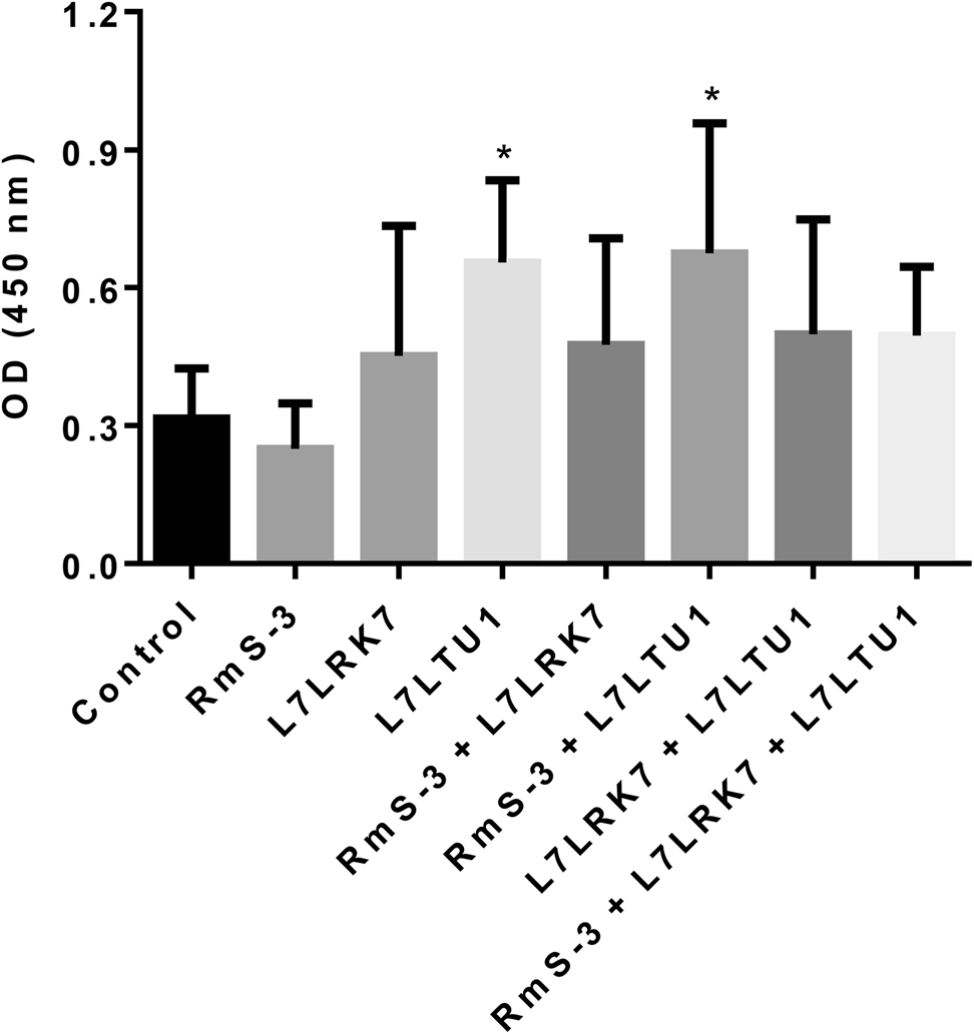
Identification results of tick salivary gland proteins by iELISA. Ordinata: absorbance value of antibody in serum combined with protein in *R. sanguineus* salivary gland; Abscissa: serum of control group and immunized group.

In order to identify whether RmS-3, L7LRK7 and L7LTU1 homologous proteins are contained in the salivary glands of *R. sanguineus*, the salivary glands of the partially engorged *R. sanguineus* were analysed by iELISA. The results (Fig. 6) showed that only the immunized groups L7LTU1 and RmS-3 + L7LTU1 showed positive in the iELISA, but RmS-3 and L7LRK7 were not detected.

### Effect of vaccination on *R. sanguineus* infestation in Kunming mice

**Figure 7:**
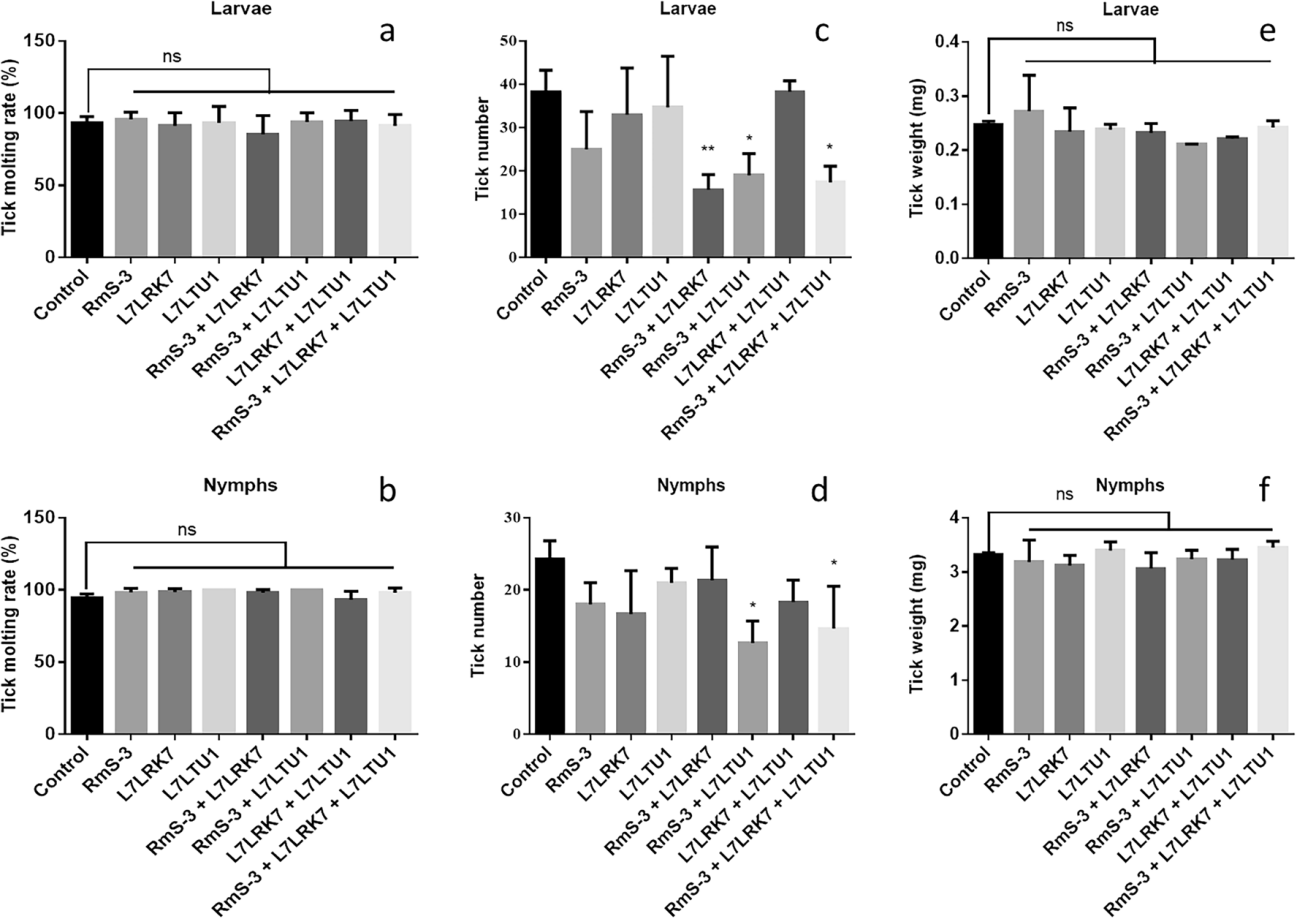
Tick number, Tick weight, and Tick molting rate in different groups after challenge. (**a**) Molting rate of tick Larvae in different groups. (**b**) Molting rate of tick Nymphs in different groups. (**c**) Number of tick Larvae in different groups. (**d**) Number of tick Nymphs in different groups. (**e**) Weight of tick Larvae in different groups. (**f**) Weight of tick Nymphs in different groups; Ordinata: Tick number, number of ticks full of blood; Tick weight, weight of ticks after full blood; Tick molting rate, ratio of molting number to full blood of ticks after fully engorged; Abscissa: different mice immunized group.

The immunized mice were challenged with *R. sanguineus* in order to compare the immunological effects of single-antigen vaccinations versus cocktail-antigen vaccines. In compared to the control group, the number of completely engorged ticks in both larval and nymphal ticks decreased. The fully engorged rate (%) of larvae and nymphs decreased to 40.87% (P < 0.05) and 87.68% (P > 0.05) in the RmS-3 + L7LRK7 immune group, 49.57% (P < 0.01) and 52.06% (P < 0.05) in the RmS-3 + L7LTU1 group, 45.22% (P < 0.05) and 60.28% (P < 0.05) in the RmS-3 + L7LRK7 + L7LTU1 immune group, as illustrated in Fig. 7. In the examined larvae and nymphs, however, there was no significant difference between groups in body weight or molting rate (P > 0.05).

## Discussion

In order to reduce the problem of increasing acaricides resistance and acaricides residue caused by the abuse of acaricides, the development of tick vaccines is considered to be an effective way. Some tick serpins have been identified as candidate targets for effective anti-tick vaccines, and they have a certain protection as single antigen vaccine^33,40–42^. nevertheless, due to the great number of tick species, the number of tick antigens with cross-protected epitopes is limited, resulting in poor broad-spectrum and generality of anti-tick vaccines^43–46^. The hunt for new antigens that are conserved among tick species can aid in the resolution of the aforementioned issues.

In this study, three serpins, RmS-3 L7LRK7, and L7LTU1 protein were selected for their high identity in *Rhipicephalus* spp. RmS-3 protein was found in salivary glands of female *R. sanguineus*, while the other two proteins, L7LRK7, and L7LTU1, were deduced by sequencing^38^. Kunming mice were inoculated with recombinant proteins RmS-3, L7LRK7, and L7LTU1, and the efficacy of the three proteins as vaccinations against *R. sanguineus* was assessed.

The tick completes its blood meal by inflicting a wound on the host’s skin and preventing healing and coagulation by secreting a number of bioactive substances in saliva^47–49^. As a consequence, avoiding anticoagulants helps to reduce tick blood feeding. The coagulation and immune protection roles of RmS-3, L7LRK7 and L7LTU1 were detected by routine blood test, coagulation reaction and T lymphocyte ratio. The APTT in the L7LTU1 and RmS-3 + L7LRK7 + L7LTU1 immunized groups was extended after the second immunization, according to the findings of the coagulation reaction. The intrinsic coagulation route is represented by APTT, while the extrinsic coagulation pathway is represented by PT. When the PT or APTT reaction time was extended, it showed that the immunological groups were more capable of inhibiting anticoagulation. As a result, the coagulation level was greater in the L7LTU1 and RmS-3 + L7LRK7 + L7LTU1 immune groups. Blood routine test results showed that there was no significant difference of RBC, HGB and PLT between the control group and the immunized after three immunizations; WBC and LYM in the L7LRK7 + L7LTU1 immunized group were significantly lower than in the control group. The percentages of CD4^+^ and CD8^+^ T lymphocytes in the RmS-3 vaccinated group decreased in the T lymphocyte subset level analysis experiment, which is consistent with the findings of Coutinho et al. which indicates that RmS-3 has a strong inhibitory effect on lymphocyte metabolic activity, IFN-production, and proliferation, but does not promote cell death^35^. Similarly, T lymphocyte subset identification findings in this experiment revealed that L7LRK7 and L7LTU1 had inhibitory effects on lymphocyte metabolic activity, which is consistent with the biological functions of RmS-3, L7LRK7, and L7LTU1 as serpins. The safety issue of immunosuppression caused by these antigens should be further evaluated in subsequent studies.

The iELISA results showed that all three proteins could stimulate high-affinity antibodies in the host, and the combination of RmS-3 + L7LRK7 and RmS-3 + L7LTU1 could produce higher affinity antibodies than the single RmS-3 and L7LTU1 proteins. Furthermore, the salivary glands of *R. sanguineus* were extracted and analyzed for the presence of these three proteins. The L7LTU1 protein can be detected by antibodies in serum. This means that the antibody can bind to the L7LTU1 protein in the salivary gland, thereby preventing its function. The other two proteins could not be detected, probably due to the absence or lower amounts in salivary glands.

Although it has been demonstrated that a cocktail of multiple antigens may be more effective^22,13^, the results of immunogenicity analysis in this study revealed that the immunogenicity of the cocktail vaccine of three antigens was lower, while the immunogenicity of the cocktail vaccine of two antigens was better. This may be explained by antigen competition and cross-immunity. Cross-immunity means that antibodies directed against a particular antigen confer immunity to another antigen. Antigen competition means that after the host is vaccinated with a cocktail, the immune response generated by one antigen will inhibit the immune response generated by the other antigen. The mechanism of this phenomenon remains unclear^50^. It has been speculated that this may be due to intramolecular or intermolecular competition between determinants of the identical or different immunogens^51^. Therefore, in this experiment, the cocktail vaccine of three antigens may produce antigen competition in the host, which reduces the amount of antibody produced. However, a cocktail of two antigens may enhance cross-immunity and increased antibody production.

Bioinformatics analysis is beneficial in the creation of vaccines. In this paper, bioinformatic investigation predicted that these three proteins have a high number of epitopes, which help to trigger the host immune response. The iELISA test revealed that all three proteins could produce high affinity antibodies in Kunming mice, correlating with the bioinformatics analysis results. Bioinformatics study also revealed that these three proteins are highly similar to those found in *R. sanguineus*. It’s speculated that these proteins provide immune protection against *R. sanguineus*. The assumption was verified in the tick challenge assay, that the immune groups RmS-3 + L7LTU1 and RmS-3 + L7LRK7 + L7LTU1 showed better immune protection, which could inhibit the feeding of larvae and nymphs. This finding, however, does not perfectly coincide with the iELISA result, indicating that antibody titers may not reflect all immune protection in vivo. While RmS-3, L7LRK7 and L7LTU1 inhibited the number of larvae and nymphs with full blood meal, further experiments are needed in adult ticks.

### Supplementary Information

Supplementary Legends.

Supplementary Figure 1.

Supplementary Figure 2.

Supplementary Figure 3.

Supplementary Figure 4.

## Data Availability

The datasets generated during and/or analysed during the current study are available from the corresponding author on reasonable request. Further inquiries can be directed to the corresponding authors.
