# Supplementary material for: Immune protection of three serine protease inhibitors vaccine in mice against Rhipicephalus sanguineus
Source: Sci Rep. 2024 Apr 2;14:7703. doi: 10.1038/s41598-024-58303-4 (PMC10987660; doi:10.1038/s41598-024-58303-4)
Supplement: Supplementary file 1 — Supplementary Legends. [file 41598_2024_58303_MOESM1_ESM.docx]

Supplementary figure 1. Surface probability, flexibility, B cell epitope and T cell epitope prediction of RmS-3, L7LRK7 and L7LTU1 proteins. **a** RmS-3; **b** L7LRK7; **c** L7LTU1.

Supplementary figure 2.Expression and purification of rRmS-3 analyzed by SDS-PAGE. lane M, protein marker; Lanes 1 and 2, the crude extract from cell; Lanes 3 and 4, the RmS-3; Lanes 6, 7, 8 and 9, chitin beads after elution.

Supplementary figure 3.Expression and purification of rL7LRK7 analyzed by SDS-PAGE. lane M, protein marker; Lanes 1, 2 and 3, The crude extract from cell; Lanes 4 and 5, the L7LRK7; Lanes 6, 7, 8 and 9, Chitin beads after elution.

Supplementary figure 4.Expression and purification of rL7LTU1 analyzed by SDS-PAGE. lane M, protein marker; Lanes 1 and 2, the crude extract from cell; Lanes 3 and 4, chitin beads after elution; Lanes 5 and 6,the L7LTU1.
