# Supplementary figures and images for: Immune protection of three serine protease inhibitors vaccine in mice against Rhipicephalus sanguineus
Source: Sci Rep. 2024 Apr 2;14:7703. doi: 10.1038/s41598-024-58303-4 (PMC10987660; doi:10.1038/s41598-024-58303-4)

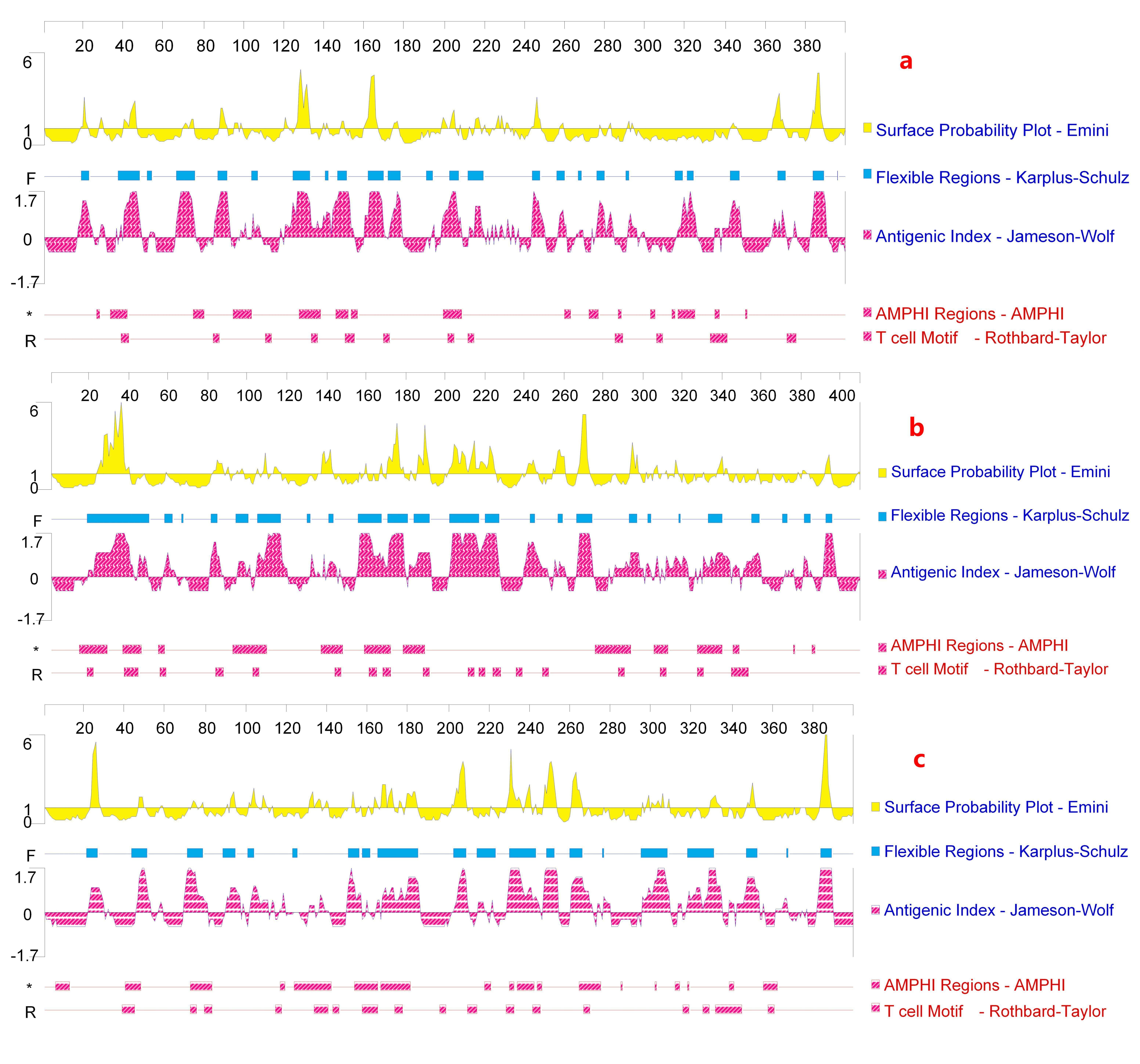

Supplement: Supplementary file 2 — Supplementary Figure 1. [file 41598_2024_58303_MOESM2_ESM.jpg]

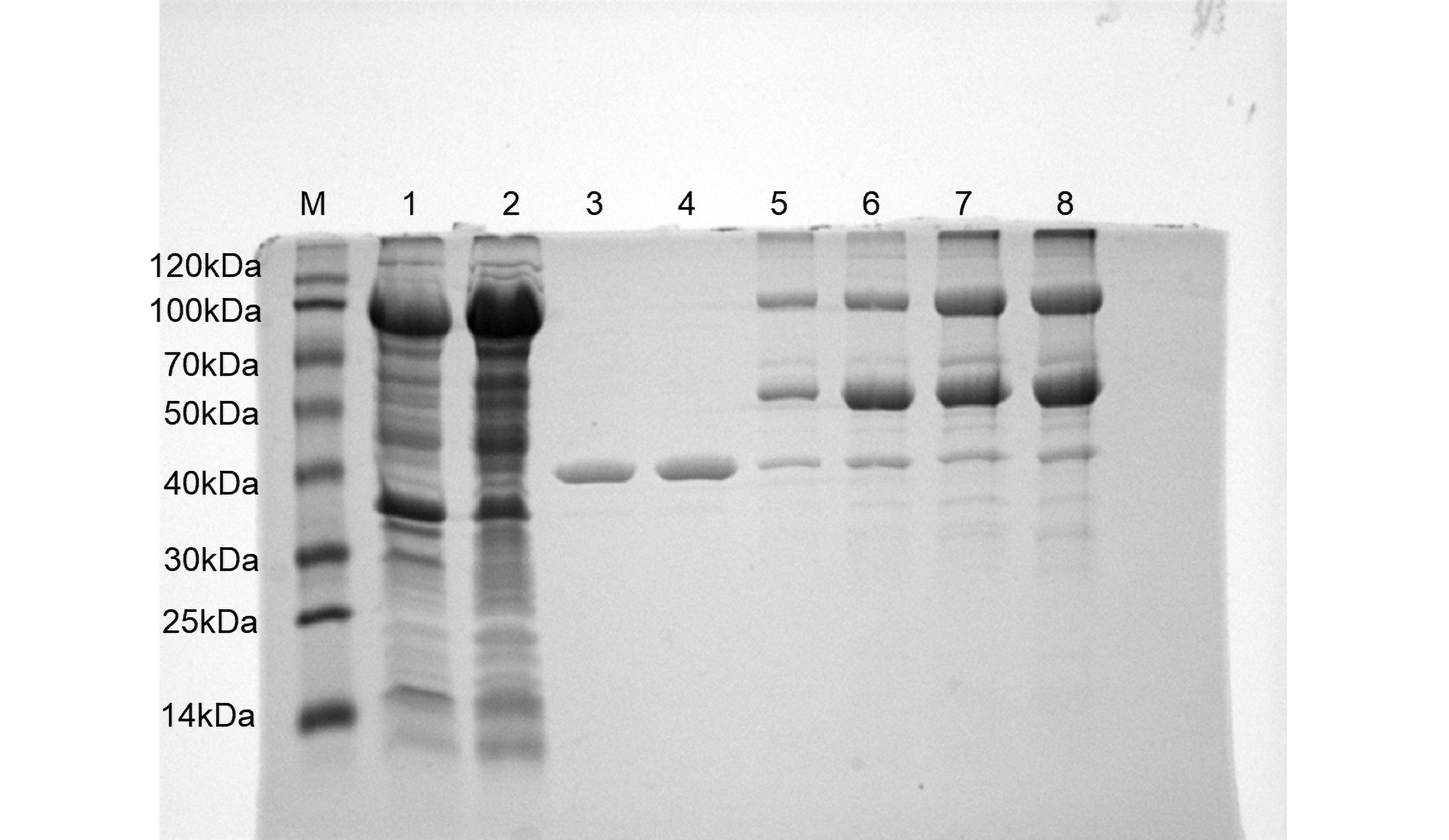

Supplement: Supplementary file 3 — Supplementary Figure 2. [file 41598_2024_58303_MOESM3_ESM.tif]

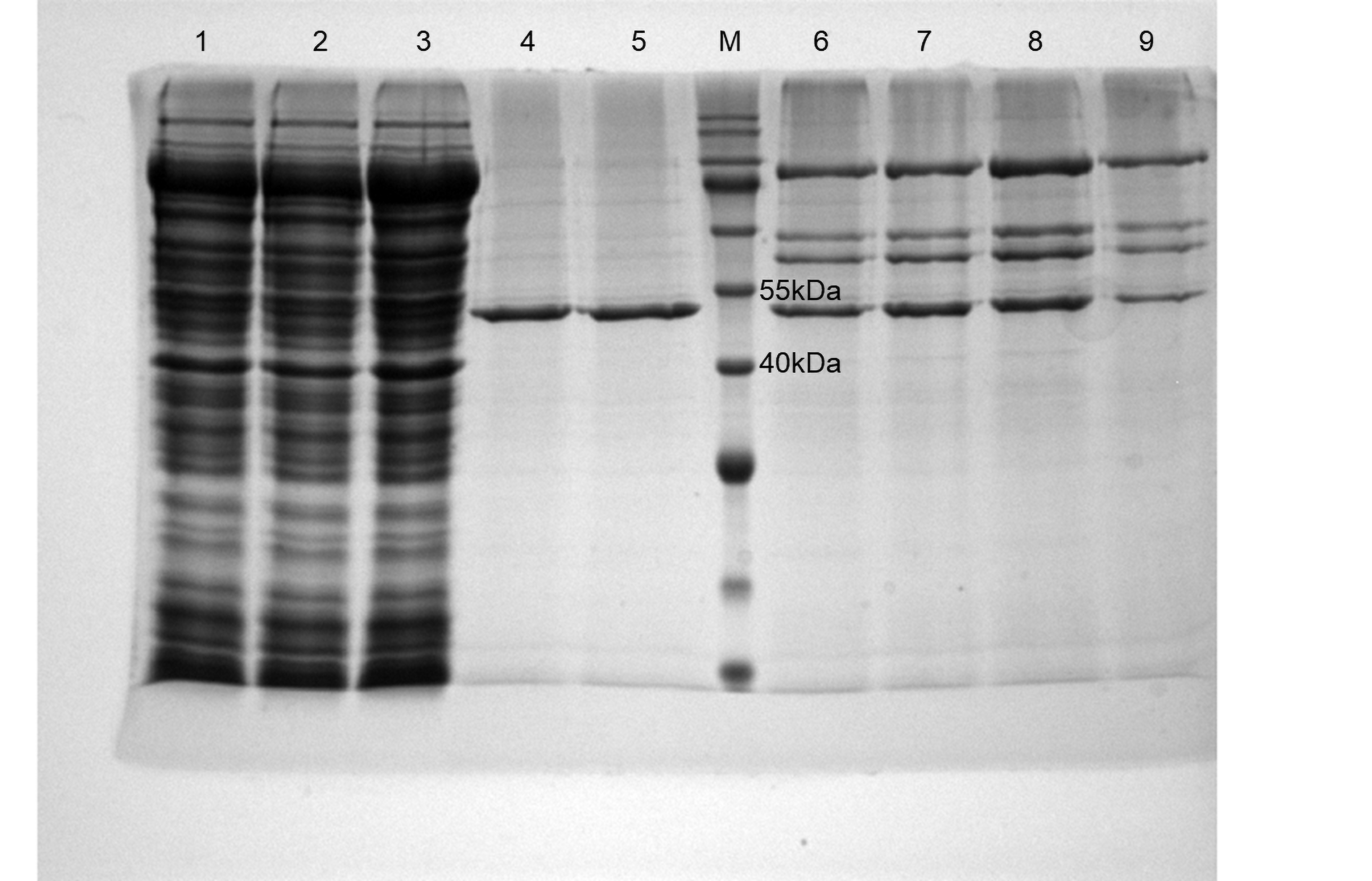

Supplement: Supplementary file 4 — Supplementary Figure 3. [file 41598_2024_58303_MOESM4_ESM.tif]

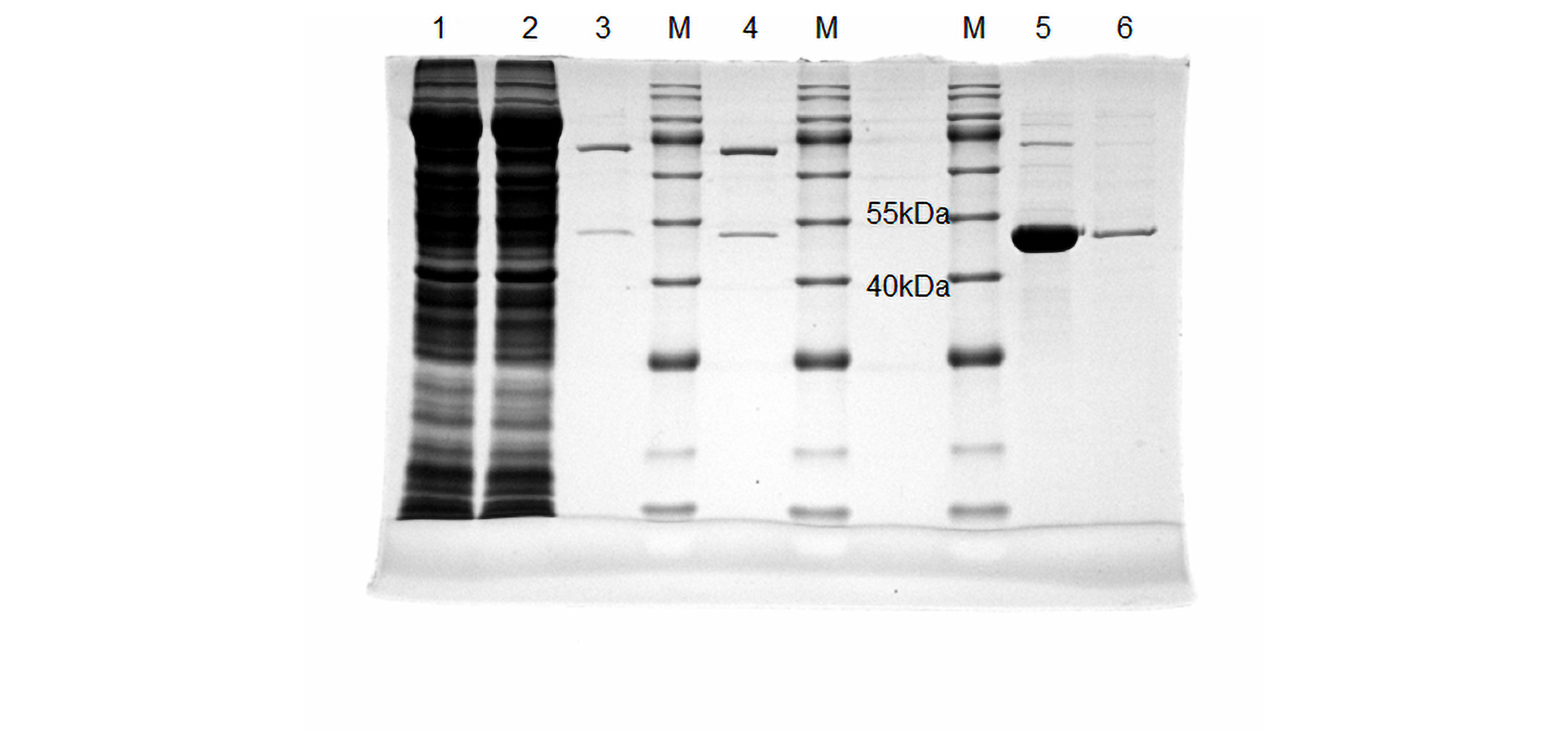

Supplement: Supplementary file 5 — Supplementary Figure 4. [file 41598_2024_58303_MOESM5_ESM.tif]
